# Breast Cancer Awareness and Screening Perceptions of Women in Yerevan, Armenia

**DOI:** 10.3389/ijph.2024.1607029

**Published:** 2024-05-16

**Authors:** Haley Tupper, Razmik Ghukasyan, Armine Bayburtyan, Marine Hovhannisyan, Shant Shekherdimian

**Affiliations:** ^1^ University of California, Los Angeles, Los Angeles, CA, United States; ^2^ Yerevan State Medical University, Yerevan, Yerevan, Armenia

**Keywords:** Armenia, low and middle income countries (LMICs), breast cancer screening, Champion’s Health Belief Model Scale (CHBMS), perception

## Abstract

**Objectives:**

Breast cancer is the leading cause of female cancer mortality in Armenia. The government is considering covering breast cancer screening, but prevailing attitudes towards it are unknown. This cross-sectional study assessed Armenian women’s awareness and perceptions of breast cancer screening.

**Methods:**

We administered a validated telephone survey to women ages 35–65 registered in Yerevan’s polyclinic system between 2019–2021, assessing sociodemographic characteristics, breast cancer exposure and screening attitudes, using an adapted Champion’s Health Belief Model Scale (CHBMS). We analyzed the association, unadjusted and adjusted, between sociodemographic characteristics, screening exposure, and CHBMS scores.

**Results:**

170 women completed surveys. Most (82.9%) were aware of screening, 48.5% knew someone with breast cancer, but only 42.5% had undergone screening, predominantly without their physician’s recommendation (63.2%). Despite elevated awareness, 76.2% had never discussed screening with their provider. Barriers included cost and mistreatment concerns. Education consistently predicted prior screening and most CHBMS scores.

**Conclusion:**

Armenian women are highly exposed to breast cancer, but knowledge and prior screening primarily emanate from non-physician sources. Results highlighted the influence of education, patient-provider relationships, and healthcare costs, underscoring the importance of multi-level interventions.

## Introduction

Breast cancer is an important cause of female morbidity and mortality worldwide. In Armenia, a former Soviet republic in the South Caucasus, breast cancer accounts for 22.6% of female cancer cases and is the leading cause of cancer-related mortality among women [1]. With an age-standardized mortality-to-incidence ratio of 0.41, breast cancer in Armenia more closely resembles breast cancer patterns in low-to-medium human development index (HDI) countries than among Armenia’s HDI peers [1, 2]. Compared to countries at a similar level of development, Armenia has nearly double the mortality from breast cancer among women ages 15–49 [3, 4]. Almost one-quarter (20.8%) of cases are diagnosed at stage IV [5] when treatment costs are typically prohibitive and 5-year survival is only 2.5 months if untreated [6]. Although non-governmental organizations and the government have piloted screening in different administrative regions of Armenia, breast cancer screening is still largely opportunistic [7]. With the introduction of universal health coverage, the Armenian Ministry of Health seeks to establish a national breast cancer screening program as part of the basic benefits package. Organized screening is an important component of reducing breast cancer morbidity and mortality, reducing mortality by 15%–20% [8, 9]. In general, screening is a health behavior that requires elective individual participation. There are many important socioecological levels to consider with regards to cancer screening. To examine the individual level, one of the most widely used social behavior frameworks is the Champion’s Health Belief Model Scale (CHBMS) [10]. This study employs a rigorously-translated and adapted Eastern Armenian version of the CHBMS to understand individual health beliefs that would influence screening uptake [11]. The objective of this study was to evaluate the individual knowledge, attitudes and beliefs of screening-age Armenian women towards breast cancer screening.

## Methods

This cross-sectional telephone-based study evaluating Armenian women’s breast cancer screening exposure, knowledge and attitudes was conducted between 2019–2021. The study design, including the consent process, underwent ethical review by both international (American) and local (Armenian) review boards (University of California, Los Angeles: Office of Human Research Protection Program: #19–001507; Yerevan State Medical University: Nº3-2/19) and was deemed exempt from full review due to minimal risk. Patient identifiers (age, gender, phone number, demographic district) were solely identified from the Ministry of Health public polyclinic registry, which was approved in the ethical review process. This study was performed in accordance with the ethical standards as established in the 1964 Declaration of Helsinki and its later amendments or comparable ethical standards.

Women ages 35–65 residing in Yerevan, Armenia who were registered in the public polyclinic system were eligible for study inclusion. All Armenian citizens are registered with a public polyclinic, irrespective of whether they choose to receive care in this system. Women with a prior diagnosis of breast cancer or surgery for breast mass were excluded. Demographic information and telephone numbers were obtained from the Ministry of Health, which maintains a list of public polyclinic registrants. Public polyclinic lists are stratified by administrative district as each polyclinic serves a distinct geographic catchment area; this stratification was maintained in the sampling strategy as each district represents distinct socioeconomic and healthcare access realities. Random sampling proportional to the eligible population in each district was performed using random number generator. Women were called by trained surveyors to undergo the survey Monday through Sunday after 5 p.m. to account for work hours and each number was attempted once to ensure consistency. Household telephone access is nearly universal (96.3% of households) in Armenia and is a common survey method for population-level cross-sectional studies [12]. Prior to conducting the survey, women were asked filter questions to ensure they met eligibility criteria and verbal consent was obtained. The surveyors then administered a previously-validated Armenian-language survey instrument to eligible, consenting participants [11]. The first half of this structured questionnaire evaluates participants’ demographics and prior exposure to breast cancer and breast cancer screening, while the second half is an adapted version of the Champion’s Health Belief Model Scale (CHBMS). The CHBMS, which integrates several health behavior theories, is a widely-used model to evaluate individual health beliefs that ultimately predict breast cancer screening behavior. The CHBMS uses a Likert-type scale (1–5) to assess five domains: 1) Perceived Susceptibility, 2) Perceived Benefits, 3) Perceived Barriers, 4) Self-Efficacy, and 5) Fear. In this scale, 1 represents completely disagree or impossible, 2 represents somewhat disagree or unlikely, 3 represents neutral, 4 represents somewhat agree or likely, and 5 represents completely agree or very likely. Individual domain questions are available in the supplement. According to research in a variety of contexts, the numeric responses in each domain differ significantly between screeners and non-screeners [10, 13, 14]. Respondents were not reimbursed for their participation.

1788 phone numbers were attempted; 716 (40.0%) of these were working numbers and 496 (27.7%) answered the phone call. 239 consenting women agreed to complete the survey. No data was collected on non-consenting individuals. Surveys that were less than 90% complete were considered to be incomplete and were removed from the analysis. Out of the 239 initiated surveys, 69 were removed due to incompleteness, resulting in 170 complete surveys for analysis.

STATA v16.1 was used for the statistical analysis. Minimum sample size calculations were performed based on the reported prevalence of prior mammography in our target age group, resulting in a minimum sample size of 161 women (2016 prevalence of prior mammography in women ages 30–60 years in Armenia: 11.9%; confidence level: 95%; margin of error: 5%) [15]. No adjustments were made for the sampling strategy in the analysis. *p*-values of 0.05 or less were considered to be statistically significant unless otherwise stated and confidence intervals are 95% confidence intervals (CI). The following analyses were performed: 1) Descriptive analysis of respondent characteristics, including prior exposure to breast cancer screening and CHBMS Likert-type responses, reporting on frequency; 2) Association analysis between demographic and breast cancer screening exposure variables, reporting on Chi-square statistic; 3) Correlation analysis between aggregated CHBMS domain responses, reporting on Spearman’s rank correlation coefficient; and 4) Multivariate linear regression, reporting on adjusted regression coefficients, and multivariate logistic regression, reporting on adjusted odds ratios. Low frequency observations for categorical variables (i.e., <10 observations) were collapsed as follows for association and regression analysis: For employment, unemployed and other were condensed into one level (unemployed/other) and for monthly expenses, the two highest expense brackets were condensed into one level (>300,000 AMD). $1 USD is approximately 385 AMD, although the exchange rate fluctuates. Of note, given the prevalence of highly traditional gender roles in Armenian society, monthly household expenses were used as a proxy for income or spending power.

For the correlation analysis and multivariate linear regression, individual CHBMS Likert-type responses (1–5) were aggregated by domain (Perceived Susceptibility, Perceived Benefits, Perceived Barriers, Self-Efficacy, and Fear) to generate a total score for each domain. Key outcomes evaluated using multivariate linear regression were the aggregate scores for each domain (susceptibility, benefits, barriers, self-efficacy, and fear). Key outcomes evaluated using multivariate logistic regression were prior knowledge of breast cancer screening, prior discussion with physician about breast cancer screening, and prior receipt of breast cancer screening imaging. For the regression analyses, potential predictor variables were selected based on domain knowledge. The following predictor variables were included in each regression model: age, insurance status (reference level: uninsured), highest education complete (reference level: high school), employment (reference level: full-time employed), estimated monthly expenses (reference level: <100,000 AMD), and having an acquaintance with breast cancer (reference level: none).

## Results

Respondents (*n* = 170) median age was 49 years and the majority of women were married, employed, religious, uninsured and had received a tertiary education with monthly expenses roughly equivalent to the monthly nominal wage *per capita* of Armenia (Table 1) [16]. These demographics are reflective of the more affluent population of Yerevan compared to rural areas based on 2015–2016 Demographic & Health Survey Data [17]. Women were highly aware of breast cancer; nearly half (48.5%, *n* = 82) knew someone who had been diagnosed with breast cancer and almost one-quarter (22.4%, *n* = 38) knew someone who had died of breast cancer. Most women had heard about a test that could detect breast cancer early (82.9%, *n* = 141) but less than a quarter had discussed breast cancer screening with their doctor (23.5%, *n* = 40). Of the 42.5% (*n* = 71) of women who had previously been screened, their doctor had initiated the screening only 20.6% (*n* = 14) of the time. Insurance status was associated with employment, where respondents who were employed full-time were more likely to have insurance (*p* = 0.02). The median amount that women would be willing to pay out-of-pocket for breast cancer screening was 7000 AMD, roughly equivalent to $18 USD, but the range was wide [10–20000 AMD ($0-$52 USD)].

**TABLE 1:** Respondent characteristics and exposure to breast cancer and screening (Yerevan, Armenia. 2019–2021).

Total	*n* = 170	%
	*n*	
Age^a^	49	41–57
Marital Status		
Married	126	74.1%
Single	21	12.4%
Divorced	10	5.9%
Widowed	13	7.7%
Highest Education Completed		
High School	32	18.8%
Vocational Degree	41	24.1%
College Graduate and Beyond	97	57.1%
Employment Status		
Full-time Employed	90	52.9%
Part-time Employed	18	10.6%
Unemployed	56	32.9%
Other	6	3.5%
Estimated Monthly Expenses		
<100,000 AMD (<$200)	31	18.7%
100–300,000 AMD ($200–600)	104	62.7%
300–500,000 AMD ($600–1,000)	28	16.9%
>500,000 AMD (>$1,000)	3	1.8%
Insurance Status		
Insured	35	20.6%
Uninsured	135	79.4%
Religiosity		
I am an atheist	2	1.2%
I am not very religious and rarely attend church	4	2.4%
I am religious but do not regularly attend church	142	84.5%
I am religious and regularly attend church	20	11.9%
Family History (Mother, Sister, Aunt, Grandmother)		
Breast Cancer	29	17.1%
Breast Cancer Mortality	13	48.2%
Communal Exposure (Non-Relative)		
Breast Cancer	82	48.5%
Breast Cancer Mortality	38	46.3%
Have you heard about diagnostics that can detect breast cancer early?		
Yes	141	82.9%
No	29	17.1%
Have you ever had imaging for breast cancer?		
Yes	71	42.5%
No	97	57.7%
Why did you participate in breast cancer screening?		
My doctor recommended and referred me to breast imaging	14	20.6%
I asked my doctor to be referred for breast imaging	11	16.2%
I obtained screening without my doctor’s recommendation	10	14.7%
A friend or relative recommended obtaining screening	3	4.4%
For other unlisted reasons	30	44.1%
Which screening modality do you prefer?		
Mammography	96	58.9%
Breast ultrasound	35	21.5%
Breast MRI	9	5.5%
Breast clinical exam by physician	18	11.0%
Breast self-examination	5	3.1%
Has your doctor discussed or recommended breast cancer screening to you?		
Yes	40	23.5%
No	130	76.5%
How much would you be willing to pay (in AMD)?^a^	7000	10–20000

^a^ Median, IQR.

Many socioeconomic variables were associated with prior exposure to breast cancer screening (Table 2). Tertiary education was most consistently associated with familiarity with breast cancer screening, specifically increased awareness of screening, increased prior physician-engaged screening discussions and increased prior screening participation. Notably, being insured was not associated with increased awareness of screening or prior screening participation. Furthermore, having an acquaintance with breast cancer was associated with an increased probability of prior discussion about breast cancer with their physician (*p* = 0.043), prior breast cancer imaging (*p* = 0.003), and prior self-breast exam (*p* = 0.007). Having a relative with breast cancer was not as clearly associated with breast cancer screening exposure.

**TABLE 2:** Socioeconomic status & breast cancer screening exposure (Yerevan, Armenia. 2019–2021).

		Total	Awareness of breast cancer screening tests		Prior breast cancer screening discussion with doctor		Prior participation in breast cancer screening	
			*n* (%)	*p*	*n* (%)	*p*	*n* (%)	*p*
Socioeconomic Position	Highest Education Completed			0.002		0.02		<0.0001
	High School	32	20 (62.5%)		3 (9.4%)		5 (15.6%)	
	Vocational Degree	41	34 (82.9%)		7 (17.1%)		13 (31.7%)	
	College Graduate and Beyond	97	87 (89.7%)		30 (30.93%)		53 (55.8%)	
	Employment Status			0.03		0.02		0.10
	Full-time Employed	90	80 (88.9%)		29 (32.2%)		44 (50.0%)	
	Part-time Employed	18	16 (88.9%)		2 (11.1%)		6 (33.3%)	
	Unemployed/Other	62	45 (72.6%)		9 (14.5%)		21 (33.9%)	
	Estimated Monthly Expenses			0.09		0.01		0.05
	<100,000 AMD (<$200)	31	22 (71.0%)		2 (6.5%)		7 (22.6%)	
	100–300,000 AMD ($200–600)	104	91 (87.5%)		25 (24.0%)		47 (46.1%)	
	>300 AMD (>$600)	31	28 (80.0%)		13 (37.1%)		17 (48.6%)	
	Insurance Status			0.99		<0.0001		0.42
	Insured	35	29 (92.9%)		17 (48.6%)		16 (48.5%)	
	Uninsured	135	112 (83.0%)		23 (17.0%)		55 (40.7%)	
Breast Cancer Proximity	Relative with Breast Cancer			0.11		0.13		0.18
	Yes	29	27 (93.1%)		10 (34.5%)		15 (53.6%)	
	No	141	114 (80.9%)		30 (21.3%)		56 (40.0%)	
	Non-Relative with Breast Cancer			0.21		0.04		0.003
	Yes	82	71 (86.6%)		25 (30.5%)		44 (54.3%)	
	No	87	69 (79.3%)		15 (17.2%)		27 (31.4%)	

Prior discussions about breast cancer with a physician and prior receipt of breast cancer screening imaging were each independently associated with an increased willingness to obtain future imaging (*p* = 0.01 and *p* < 0.0001, respectively). Notably, 63.2% of women obtained screening without their physician’s involvement, which may explain why factors that were associated with discussion with a physician were not also associated with prior screening participation.

**FIGURE 1:**
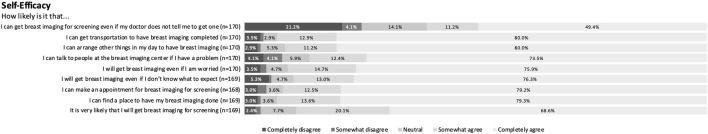
Sample set of full survey responses for self-efficacy domain (Yerevan, Armenia. 2019–2021).

CHBMS domain responses were relatively consistent across respondents with narrow variation. Respondents felt moderately susceptible to breast cancer and almost uniformly perceived breast cancer screening as beneficial. Within the barriers domain with 24 items, respondents generally disagreed with most potential barriers with only a median aggregate score of 28.5. Cost, however, was viewed as a barrier. Among respondents, 18.2% (*n* = 31) partially or completely agreed that “Not being able to afford the cost would prevent me from getting breast imaging” and 14.1% (*n* = 24) agreed that “I am reluctant to undergo breast imaging because if cancer is detected, I am afraid that I will not able to pay for my treatment.” Concern about mistreatment by healthcare workers at the imaging centers was another more prevalent barrier with 19.5% (*n* = 33) agreeing that “being treated rudely at the imaging centers would prevent me from getting breast imaging.” Respondents generally reported high self-efficacy, although they were less confident they could obtain breast cancer screening without their physician’s recommendation (Figure 1). Finally, respondents were moderately fearful of breast cancer. See the Supplemental Figure S1 for full survey responses for all domains.

Aggregate scores in one domain were weakly to moderately correlated with aggregate scores in all other domains (Table 3) [18]. In sum, higher susceptibility, barriers and fear scores all correlated with lower benefits and self-efficacy scores.

**TABLE 3:** Correlation analysis between aggregate scores for each domain (Yerevan, Armenia. 2019–2021).

Domain	Possible range	Aggregate score	Correlation between domains				
		Median (IQR)	1	2	3	4	5
1. Susceptibility	4–20	11 (4–12)					
2. Benefits	4–20	18.5 (16–20)	−0.3				
3. Barriers	24–120	28.5 (24–40)	0.4	−0.5			
4. Self-Efficacy	9–45	42 (37–45)	−0.3	0.5	−0.6		
5. Fear	6–30	14 (9–22)	0.3	−0.3	0.4	−0.2	

Multiple linear regression highlighted several important predictor variables, particularly education (reference level: high school education) and monthly expenses (reference level: <100,000 AMD). College-education predicted a lower susceptibility score by 3.26 points (CI: −5.02 to −1.51, *p* < 0.0001), and knowing someone with breast cancer predicted an increased susceptibility score by 1.63 points (CI: 0.38–2.88, *p* = 0.01). Increased monthly expenses also predicted a higher benefits score where monthly expenses of 100–300,000 AMD predicted an increased benefits score by 2.05 points (CI: 0.63–3.46, *p* = 0.005) and monthly expenses of over 300,000 AMD predicted an increased benefits score by 2.35 points (CI: 0.62–4.08, *p* = 0.008). Within barriers, education and income were again relevant predictors. College education predicted a lower barriers score by 9.71 points (CI: −16.68 to −2.74, *p* = 0.007) and monthly expenses of 100–300,000 AMD predicted a decreased barriers score by 9.01 points (CI: −16.08 to −1.95, *p* = 0.01). Within self-efficacy, college education predicted an increased self-efficacy score by 2.80 points (CI: 0.27–5.33, *p* = 0.03) and monthly expenses of 100–300,000 AMD predicted an increased self-efficacy score by 2.69 points (CI: 0.13–5.25, *p* = 0.04). No potential predictor variables were statistically significant for aggregate fear scores.

Multiple logistic regression highlighted additional predictor variables for 1) Respondent knowledge of breast cancer screening, 2) Prior discussions with physician about breast cancer screening, and 3) Prior breast cancer screening. Women who were college-educated had 4.56 times the odds of prior awareness of breast cancer screening (CI: 1.54–13.49, *p* = 0.006). Insured patients had 5.00 times the odds of prior discussions about screening with their physician (CI: 1.95–12.82, *p* = 0.001) and women who knew someone with breast cancer had 2.82 times the odds of prior breast cancer screening discussions with their physician (CI: 1.22–6.55, *p* = 0.02). Finally, women who were older were also slightly more likely to have had prior breast cancer screening with 1.07 times the odds with each additional year of age (CI: 1.02–1.12, *p* = 0.008). Women who were college-educated had 6.84 times the odds of prior screening (CI: 2.18–21.50, *p* = 0.001) and women who knew someone with breast cancer had 3.42 times the odds of prior screening (CI: 1.66–7.07, *p* = 0.001).

## Discussion

Organized screening will be an important component of addressing elevated breast cancer mortality rates in Armenia, where one-third of breast cancer diagnoses are Stage III, IV or unknown (34.6%) [5]. Our results highlight that middle-aged women in Yerevan, Armenia are highly exposed to breast cancer, but most knowledge and prior screening experiences emanate from non-physician sources (e.g., the community). Nearly half of respondents had an acquaintance with breast cancer and concurrent exposure to breast cancer mortality was also high; almost half of affected relatives and acquaintances with breast cancer had died from their cancer, consistent with Armenia’s reported mortality-to-incidence ratio of 0.41 [1]. Meanwhile, the overwhelming majority had already heard of imaging that could detect early breast cancer, but despite widespread awareness of breast cancer and screening, few women were discussing it with their doctor. Among those who had prior breast cancer screening, most had obtained screening without their physician’s involvement. Further discussion in particular is warranted on three emergent themes, specifically the impact of advanced education on female empowerment, the role of providers and the patient-provider relationship, and the cost of care. Ultimately, this trifecta highlights the essentiality of multi-level interventions for breast cancer screening.

Multiple socioeconomic factors were associated with prior clinical discussions about breast cancer screening, but education was the most salient factor in prior screening *receipt*. Education’s impact on screening attitudes (susceptibility, benefits, barriers, self-efficacy) was evident in adjusted regression analysis as well. Other socioeconomic factors, while relevant, were less consistently important. Higher susceptibility, barriers and fear scores all correlated with lower benefits and self-efficacy scores; this trend may reflect respondents’ individual agency. Although individual agency undoubtedly impacts breast cancer screening perceptions, it transcends this subject arena. In Armenia, higher education for women may be a particularly important means of enhancing their agency beyond spending power, employment and health insurance with positive effects extending beyond breast cancer and healthcare.

Domain responses also indicate that physician recommendations are important but few are recommending screening. Results reveal a guarded relationship with the medical system more broadly, where many respondents endorsed concerns about mistreatment at the imaging center as a potential screening barrier. In Armenia’s traditionally paternalistic patient-provider relationship, conversations may be relatively unidirectional and patients may not feel empowered to ask questions. Education may play a key role here as well, empowering women to pursue a more bidirectional therapeutic relationship. If Armenia establishes an organized nation-wide breast cancer screening program, it will be important for the continuing medical education system to not only reinforce breast cancer screening and management as an important component of routine care, but also to emphasize provider empathy and shared decision making in the patient-provider relationship.

Cost is a notable barrier to both cancer screening and treatment, as reflected by both responses and known realities in Armenia. Cost may be one reason providers are not routinely discussing screening. Breast cancer screening is not currently covered outside of pilot programs and non-governmental organization-driven campaigns. The basic benefits package, which covers all citizens, covers some breast cancer treatment after diagnosis but arbitrarily caps coverage at 150,000 or 300,000 dram ($388 or $776 USD), depending on social welfare category. Anecdotally, waiting lines for publicly-covered treatment are long. The ethics of screening without access to proper treatment is questionable.

The results highlight the necessity of multi-level breast cancer screening interventions. Preventive care is not a strongly engrained facet of the Armenian healthcare system and the patient-provider relationship is relatively entrenched [19]. Both components need to be prudently addressed. Our research highlights that educational breast cancer screening campaigns would need to target both providers and patients, and additional effort is needed to reach less-educated women. Aggregate and individual domain responses can be used to design tailored interventions [10]. If breast cancer screening were to be publicly funded, new basic benefits package screening coverage would need to be broadly advertised to avoid misinformation.

### Limitations

The most notable limitation in our study was the low overall response rate and relatively high incomplete response rate, which may limit the study’s generalizability and introduce bias into the study results. The low response rate may be partially due to the use of telephone-based surveys, which are a common method for cross-sectional population-level surveys but can yield low response rates. Women who fully completed the survey may be different than non-responders and partial-responders in important, unmeasured ways. The study results may only be generalizable to urban Armenian women. The relatively high educational levels and household expenditures of survey respondents are more reflective of Yerevan’s socioeconomic environment. Many results, from prior screening exposure to individual domain responses (e.g., barriers such as travel), may differ in rural women. Finally, although a validated survey instrument was employed, respondents’ interpretation of a few questions may have been ambiguous, such as insurance status, as Armenia has a universal (narrow) basic benefits package with varying benefits for different demographic groups, social insurance for select government employees, and limited private insurance, and any combination of these options may have been reported by respondents as insurance [20, 21].

### Next Steps

These results can be used to guide tailored patient-facing public health messaging about breast cancer screening. Results suggest that further research is needed to guide provider-focused messaging. Provider-focused messaging needs to promote both prevention and screening and patient agency in the patient-provider relationship. A human-centered design approach with involvement of the target audience could be useful to guide breast cancer screening campaigns for patients and providers alike.

Expansion of breast cancer screening would need to be met with expansion of appropriate treatment access. Many patients currently forgo necessary care due to high out-of-pocket costs [22]. In line with this, the government is actively assessing breast cancer treatment costs experienced by the patient to understand the adequacy of current treatment coverage. In addition to financial protection, the health system must consider access to appropriate treatment in the setting of screening. Screening serves to identify clinically non-palpable masses so pre-operative image-guided localization of masses via needle, wire or clip would become an important component of appropriate treatment. Currently, this is not a routine part of breast cancer surgery in Armenia. Expansion of access to this service would need to be carefully considered to avoid high rates of non-therapeutic lumpectomies in non-palpable, screen-identified cancers. Further research is also needed on other reported access issues such as prolonged treatment wait times, unequal geographic access, chemotherapy stock-outs, and a robust chemotherapeutic black market of uncertain safety and efficacy. Breast cancer screening considerations cannot be separated from considerations about the availability of appropriate treatment.

### Conclusion

Women in Armenia are highly aware of breast cancer and screening, but rarely discuss the subject with their physician. In this survey, most prior screening experiences were not physician-initiated. Education played a particularly important role in respondent agency with regards to breast cancer and screening campaigns may need to be tailored towards less-educated women. The impact of provider recommendations, patient-provider relationships and cost cannot be neglected in any breast cancer screening program. Multi-level interventions are needed to make breast cancer screening widely accessible and accepted in Armenia.

## Supplementary Material

SUPPLEMENTARY FIGURE S1 Perceived Susceptibility, Perceived Benefits, Perceived Barriers & Fear (Yerevan, Armenia. 2019-2021).
