# Supplementary figures and images for: Breast Cancer Awareness and Screening Perceptions of Women in Yerevan, Armenia
Source: Int J Public Health. 2024 May 16;69:1607029. doi: 10.3389/ijph.2024.1607029 (PMC11137264; doi:10.3389/ijph.2024.1607029)

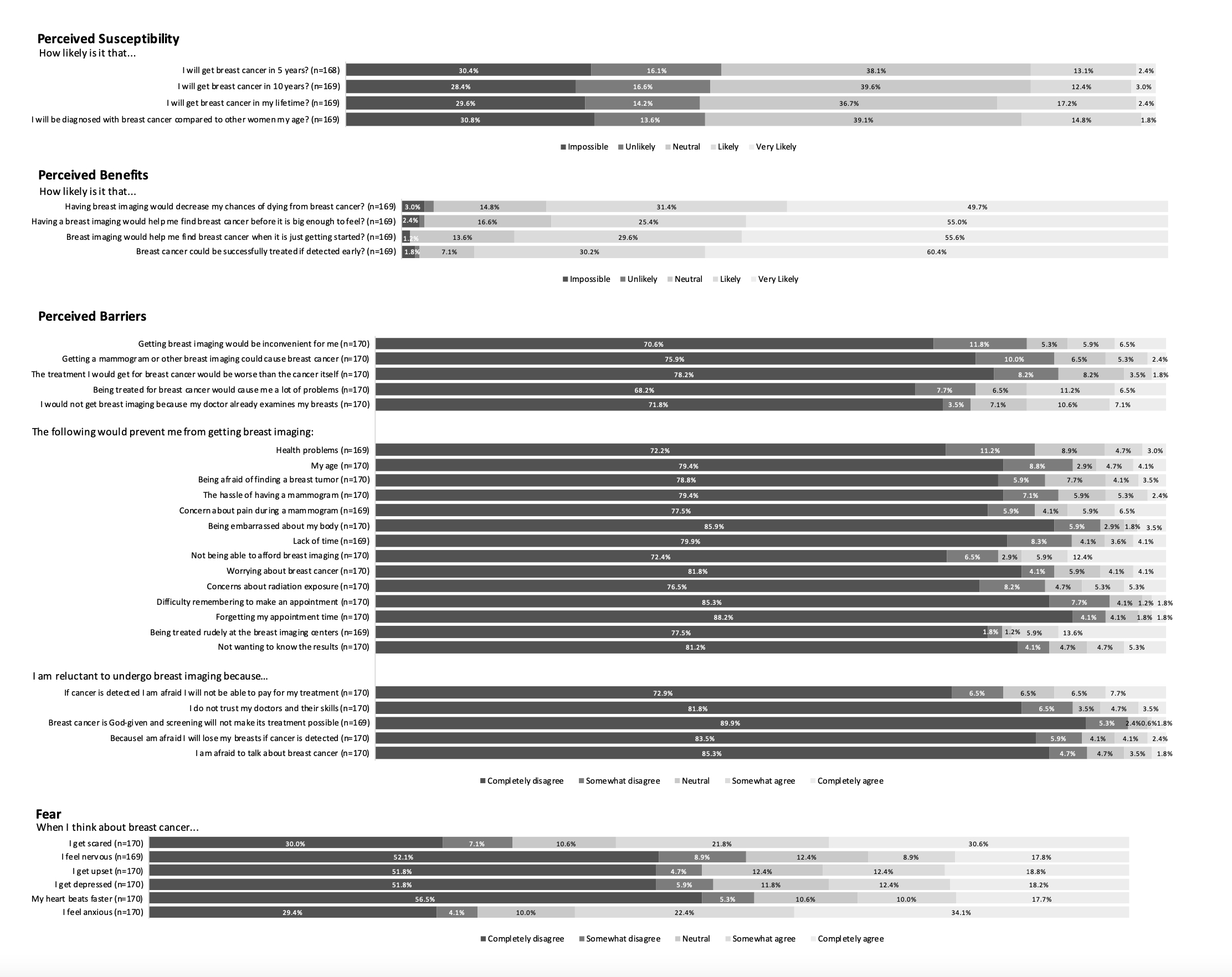

Supplement: Supplementary file 1 [file Image1.JPEG]
